# ^13^C relaxation experiments for aromatic side chains employing longitudinal- and transverse-relaxation optimized NMR spectroscopy

**DOI:** 10.1007/s10858-012-9650-5

**Published:** 2012-07-03

**Authors:** Ulrich Weininger, Carl Diehl, Mikael Akke

**Affiliations:** Center for Molecular Protein Science, Department of Biophysical Chemistry, Lund University, P.O. Box 124, 22100 Lund, Sweden

**Keywords:** Relaxation, Protein dynamics, Aromatic side chain, Sensitivity enhancement, TROSY

## Abstract

Aromatic side chains are prevalent in protein binding sites, perform functional roles in enzymatic catalysis, and form an integral part of the hydrophobic core of proteins. Thus, it is of great interest to probe the conformational dynamics of aromatic side chains and its response to biologically relevant events. Indeed, measurements of ^13^C relaxation rates in aromatic moieties have a long history in biomolecular NMR, primarily in the context of samples without isotope enrichment that avoid complications due to the strong coupling between neighboring ^13^C spins present in uniformly enriched proteins. Recently established protocols for specific ^13^C labeling of aromatic side chains enable measurement of ^13^C relaxation that can be analyzed in a straightforward manner. Here we present longitudinal- and transverse-relaxation optimized pulse sequences for measuring *R*_1_, *R*_2_, and {^1^H}–^13^C *NOE* in specifically ^13^C-labeled aromatic side chains. The optimized *R*_1_ and *R*_2_ experiments offer an increase in sensitivity of up to 35 % for medium-sized proteins, and increasingly greater gains are expected with increasing molecular weight and higher static magnetic field strengths. Our results highlight the importance of controlling the magnetizations of water and aliphatic protons during the relaxation period in order to obtain accurate relaxation rate measurements and achieve full sensitivity enhancement. We further demonstrate that potential complications due to residual two-bond ^13^C–^13^C scalar couplings or dipolar interactions with neighboring ^1^H spins do not significantly affect the experiments. The approach presented here should serve as a valuable complement to methods developed for other types of protein side chains.

**Electronic supplementary material:**

The online version of this article (doi:10.1007/s10858-012-9650-5) contains supplementary material, which is available to authorized users.

## Introduction

Protein dynamics plays a key role in protein function, including ligand binding, enzyme catalysis, and signal transduction. NMR spectroscopy is a powerful technique for studying such dynamic processes with high resolution across a wide range of time scales, extending from picoseconds to seconds or longer (Palmer 2004; Igumenova et al. 2006). The majority of studies published to date have focused on backbone dynamics through measurement of ^15^N spin relaxation parameters (Jarymowycz and Stone 2006), but the critical role of side chains in mediating protein function has spawned the development of relaxation methods for methyl groups (Palmer et al. 1993; Muhandiram et al. 1995; Ishima et al. 2001; Millet et al. 2002), methylene groups (Yang et al. 1998), side-chain amides (Boyd 1995), carboxylates/carbonyls (Paquin et al. 2008), secondary amines of arginine and tryptophan (Berglund et al. 1995), and primary amines of lysines (Iwahara et al. 2007).

Aromatic residues occur frequently in the binding interfaces of proteins (Lo Conte et al. 1999). In particular, Tyr and Trp are overrepresented in “hot spots” that contribute a large fraction of the binding free energy (Bogan and Thorn 1998), Tyr is prevalent in antigen-binding sites of antibodies (Lo Conte et al. 1999; Birtalan et al. 2010), and His and Tyr play prominent roles in enzyme catalysis (Bartlett et al. 2002). Thus, it is of great interest to monitor the dynamics of aromatic side chains and changes in this dynamics upon formation of biologically relevant complexes. Furthermore, aromatic residues constitute a significant volume fraction (roughly 25 %) of the protein interior, and therefore represent an attractive complement to methyl-containing residues (which correspond to ca. 50 % of the core) as probes of the dynamics of the hydrophobic core (Wüthrich and Wagner 1975). Previous applications of ^13^C relaxation have indicated that aromatic side chains show a rich variation in dynamics (Palmer et al. 1993), and theoretical considerations have predicted that ^13^C relaxation rates should be quite sensitive to the motions of aromatic side chains (Levy and Sheridan 1983).

Relaxation experiments on uniformly ^13^C-enriched aromatic side chains are seriously hampered by the strong *J*-coupling between neighboring sites in the aromatic rings. Labeling using 1-^13^C_1_- or 2-^13^C_1_-glucose solves the problem by creating isolated ^13^C sites in aromatic side chains, i.e. sites that do not have any ^13^C–^13^C one-bond couplings (Teilum et al. 2006; Lundström et al. 2007). Specifically, labeling with 1-^13^C_1_-glucose introduces isolated ^1^H–^13^C pairs at the Cδ positions of Phe and Tyr, the Cδ1 and Cε3 of Trp, and the Cδ2 and Cε1 of His, while 2-^13^C_1_-glucose yields ^13^C at the Cε positions of Phe and Tyr, and the Cζ3 and Cζ2 of Trp. Thus, the approach results in two complementary labeling patterns of isolated ^13^C–^1^H spin pairs suitable for canonical inverse-detected heteronuclear relaxation experiments similar to those developed for ^15^N spins (Teilum et al. 2006; Boyer and Lee 2008; Sapienza et al. 2011).

However, it is evident that relaxation studies of aromatic ^13^C spin systems can benefit from further pulse sequence optimization to achieve higher sensitivity. Specifically ^13^C-labeled protein samples produced using ^13^C_1_-glucose are limited in sensitivity by the resulting incorporation level of 50 % (Teilum et al. 2006; Lundström et al. 2007). The inherently fast transverse relaxation of aromatic ^13^C spins, due in part to their sizeable chemical shift anisotropy (CSA), further reduces the relative sensitivity of these experiments; for example, *R*_2_ is roughly a factor of 4 greater than that of backbone ^15^N spins. Furthermore, His and Trp residues contain exchangeable protons vicinal to the ^1^H–^13^C pairs of interest; chemical exchange of these protons with water might cause differential effects on the intensities of the ^1^H–^13^C cross-peaks as a function of the relaxation delay, unless special care is taken to maintain the equilibrium magnetization of water throughout the relaxation period.

Here we present pulse sequences for aromatic ^13^C relaxation experiments (*R*_1_, *R*_2_ and steady-state {^1^H}–^13^C *NOE*) that include longitudinal-relaxation optimization (Pervushin et al. 2002) and either TROSY (Pervushin et al. 1997, 1998) or PEP-HSQC approaches (Palmer et al. 1991; Kay et al. 1992). As part of these developments, we also demonstrate that neither the two-bond *J* couplings between ^13^C spins, nor the dipolar interactions with remote or vicinal protons, adversely affect the relaxation data. The pulse sequences were tested on the carbohydrate binding domain of galectin-3 (Gal3C), which contains all four types of aromatic side chains: Phe, Tyr, Trp, and His. The experiments presented here significantly improve the sensitivity and overall performance compared to ‘standard’ experiments, enabling robust and efficient investigation of aromatic side chain relaxation.

## Materials and methods

### Expression and purification

The galectin-3 carbohydrate recognition domain (Gal3C; amino acid residues 113–250) was expressed and purified as described previously (Diehl et al. 2009, 2010) using M9 minimal medium containing either 100 % 1-^13^C_1_-glucose, or 50 % 1-^13^C_1_-glucose + 50 % ^12^C_6_-glucose as the sole carbon source. In addition, one partially deuterated sample was produced using M9 minimal medium containing 100 % 1-^13^C_1_-glucose in 60 % D_2_O.

### NMR experiments

All NMR spectra were recorded on a Varian DirectDrive 500 MHz spectrometer at 298 K using 0.8 mM Gal3C samples in 5 mM HEPES pH 7.4 and 7 % D_2_O. Shaped pulses were created using Pbox. Excitation and flip-back ^1^H EBURP2 pulses (Geen and Freeman 1991) had a bandwidth of 3,300 Hz (6.6 ppm) and offset of −1,400 Hz (−2.8 ppm) from the water resonance. ^1^H i-SNOB-5 pulses (Kupce et al. 1995) had a bandwidth of 1,350 Hz (2.7 ppm) and offset of 1,250 Hz (2.5 ppm), and ^13^C REBURP pulses (Geen and Freeman 1991) had a bandwidth of 5,000 Hz (40 ppm). The ^1^H carrier was set on the water frequency, while the ^13^C carrier was centered in the aromatic region. For non-L-optimized control experiments the ^1^H EBURP2 pulses were omitted, the ^1^H i-SNOB-5 pulses were replaced by rectangular hard pulses, and the water resonance was suppressed using a 3–9–19 WATERGATE sequence (Sklenar et al. 1993).

^1^H decoupling was applied during the relaxation delays using a series of ^1^H i-SNOB-5 pulses. In the *R*_1_ experiment, these pulses were spaced 50 ms apart, while in the *R*_2_ experiment they were applied in the center of an 8 ms CPMG block consisting of 8 refocusing pulses that were phase cycled as described previously (Yip and Zuiderweg 2004). Proton saturation in the {^1^H}–^13^C *NOE* experiment was achieved using a train of 180° pulses spaced by 20 ms delays (Ferrage et al. 2008, 2009). Relaxation data were acquired by interleaving relaxation delays and *t*_1_ time points.

### Data analysis

Spectra were processed using NMRpipe (Delaglio et al. 1995) and analyzed using NMRview (Johnson and Blevins 1994). Errors in the peak intensitites were estimated from the baseplane noise and duplicate data points. Relaxation data were fittted to mono-exponential decays. Errors in the fitted relaxation rate constants were estimated from the covariance matrix of the Levenberg–Marquardt fitting.

## Results and discussion

### Pulse sequences

In an effort to optimize the sensitivity of aromatic ^13^C relaxation experiments, we implemented improved pulse sequences for the measurement of the longitudinal relaxation rate (*R*_1_), the transverse relaxation rate (*R*_2_), and the {^1^H}–^13^C *NOE*, which commonly form the basis for spectral density mapping or model-free analysis of protein dynamics.

**Fig. 1:**
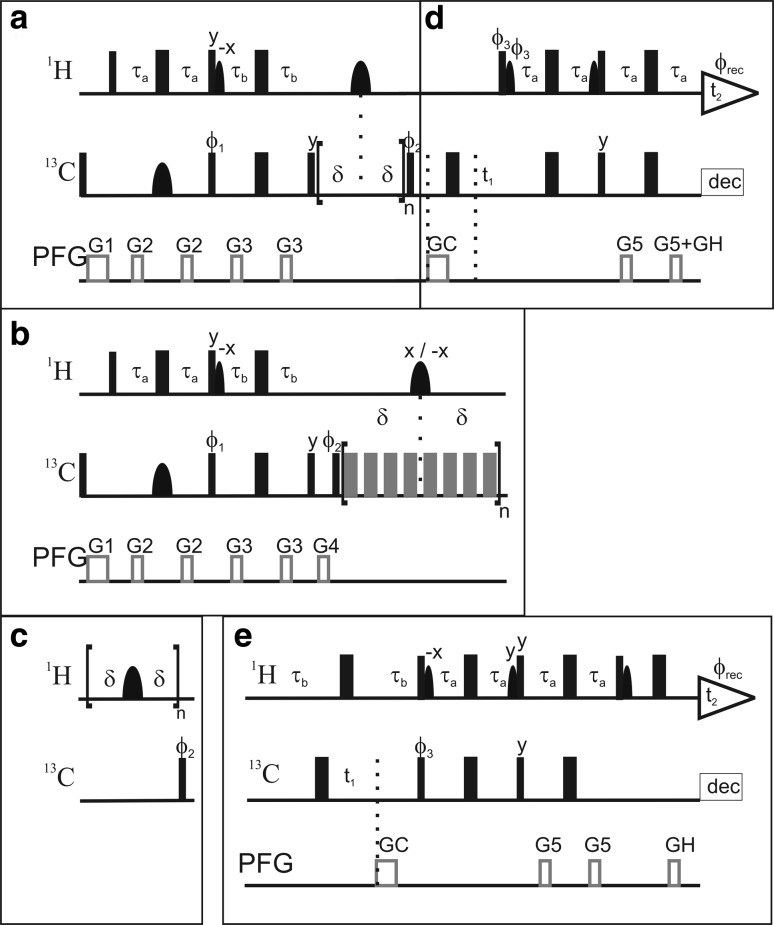
L-optimized pulse sequences for measuring aromatic ^13^C relaxation parameters. The pulse sequence of the *R*_1_ relaxation experiment shown in the *top panel* is divided into two blocks that encode: **a** polarization transfer from ^1^H to ^13^C and the relaxation period; and **d** the ^13^C evolution period followed by polarization transfer back to ^1^H. The *R*_2_ experiment is obtained by replacing *block a* with *block b*, and similarly the {^1^H}–^13^C *NOE* experiment is given by *block c*. The TROSY transfer sequence of *block d* can be substituted for the PEP-HSQC sequence given in *block e.* Each of the three relaxation experiments specified by *blocks a–c* can be combined with either of *blocks d* or *e*. *Narrow (wide) filled bars* represent 90° (180°) rectangular high power pulses. *Grey wide bars* in *block b* represent 180° rectangular CPMG pulses attenuated by 6 dB compared to the other hard pulses. *Filled bell-shaped bars* represent shaped pulses. *Narrow bell-shaped bars* on ^1^H represent EBURP2 shapes (bandwidth of 6.6 ppm, shifted 2.8 ppm upfield), while *wide bell-shaped bars* are i-SNOB-5 pulses (bandwidth of 2.7 ppm, shifted 2.5 ppm downfield). *Wide bell-shaped* pulses on ^13^C represent REBURP shapes (bandwidth 40 ppm). Pulsed field gradients (PFG) are indicated as *grey open bars*. Phases are *x* unless otherwise indicated. In all sequences τ_a_ = 1.5 ms and τ_b_ = 1.623 ms. The delay δ varies between blocks: in *a*, δ = 25 ms; in *b*, δ = 4 ms; and in *c*, δ = 10 ms. In all experiments echo/anti-echo selection were made during t_1_ by reversing ϕ_3_, GC, and the even-numbered increments of ϕ_rec_. For every second t_1_ increment ϕ_2_ and ϕ_rec_ were incremented. Durations and strengths of the gradients are G1 = (1 ms, 10 G/cm); G2 = (0.5 ms, 8 G/cm); G3 = (0.5 ms, 12 G/cm); G4 = (0.5 ms, 16 G/cm), G5 = (0.5 ms, 18 G/cm), GC = (1 ms, −50 G/cm), GH = (0.5 ms, 25 G/cm). The phase cycling for the different experiments is: a + d and b + d, ϕ_1_ = (x, x, x, x, −x, −x, −x, −x), ϕ_2_ = (y, x, −y, −x), ϕ_rec_ = (x, −y, −x, y, −x, y, x, −y); c + d, ϕ_2_ = (y, x, −y, −x), ϕ_rec_ = (x, −y, −x, y); a + e and b + e, ϕ_1_ = (x, x, −x, −x), ϕ_2_ = (y, −y), ϕ_rec_ = (x, −x, −x, x); c + e, ϕ_2_ = (y, −y), ϕ_rec_ = (x, −x). The phase cycling within the CPMG block is (x, x, y, −y, x, x, −y, y) in b + e, and (y, y, −x, x, y, y, x, −x) in b + d. The phase of the shaped ^1^H pulse in middle of the CPMG block is x for CPMG block n and −x for n + 1

The pulse sequences for the *R*_1_, *R*_2_, and {^1^H}–^13^C *NOE* experiments are shown in Fig. 1. Each relaxation experiment was implemented in the framework of either ^1^H–^13^C PEP-HSQC or ^1^H–^13^C TROSY-HSQC spectra (Fig. 1d–e). The INEPT transfer delays were tuned to a ^1^H–^13^C coupling constant of ^1^ *J*_HC_ = 155 Hz, which yields near-optimal transfer for most aromatics, except for the Cε1 position of His, which has ^1^ *J*_HC_ > 200 Hz.

Longitudinal relaxation optimization (L-optimization) involves maintaining the water and aliphatic magnetizations along the +z axis whenever possible (see further below). This was achieved by implementing selective flip-back of the water and aliphatic ^1^H spins using EBURP2 pulses (Geen and Freeman 1991), as previously applied in the context of the L-GFT-TROSY experiment for aromatic rings (Eletsky et al. 2005). Furthermore, the selective ^13^C REBURP pulse (Geen and Freeman 1991) in the first INEPT transfer step refocuses only the aromatic carbons to prevent the evolution of ^1^H–^13^C couplings of aliphatics in the first INEPT and make possible the re-alignment all aliphatic ^1^H magnetization back to +z along with the water. The 180° pulses on ^1^H during the relaxation delays of the *R*_1_ and *R*_2_ experiments and the saturation pulse train of the *NOE* experiment were implemented as i-SNOB-5 pulses (Kupce et al. 1995) selective for the aromatic region.

In the *R*_2_ experiment (Fig. 1b) an additional gradient (G4) was added before the CPMG block, in order to purge non-refocused magnetization arising as a consequence of the non-uniform ^1^H–^13^C coupling constants in aromatic side chains. Off-resonance artefacts of the CPMG refocusing pulses were suppressed by the phase cycle proposed by Yip and Zuiderweg (2004). ^1^H saturation in the {^1^H}–^13^C *NOE* experiment was achieved using 180° pulses as described (Ferrage et al. 2008, 2009). TROSY selection (Fig. 1d) was implemented in the ^13^C dimension only (Pervushin et al. 1998), which allows for a simplified TROSY scheme (Eletsky et al. 2005). In the following we describe key aspects of these pulse sequences.

### TROSY versus PEP-HSQC detection in specifically ^13^C-labeled proteins

TROSY detection of aromatic resonances has been shown to be advantageous in experiments developed for uniformly ^13^C-labeled proteins (Pervushin et al. 1998). Calculations based on the chemical shift tensor of benzene (σ_11_ = 225 ppm, σ_22_ = 149 ppm, and σ_33_ = 15 ppm; Veeman 1984) indicate that the TROSY effect is close to optimal at a static magnetic field strength of 14.1 T, and provides significant sensitivity enhancement at field strengths from 11.7 to 18.8 T (Pervushin et al. 1998). The chemical shift tensors of tryptophan Cδ1 (σ_11_ = 202, σ_22_ = 121, and σ_33_ = 48 ppm; Separovic et al. 1991) and Cε3 (σ_11_ = 208, σ_22_ = 137, and σ_33_ = 15 ppm; Separovic et al. 1999) are comparable to that of benzene, indicating similar TROSY effects for these sites.

Experiments for uniformly ^13^C-labeled samples include a constant-time evolution period (17.6 ms) to refocus one-bond ^13^C–^13^C couplings (Pervushin et al. 1998), which incurs a significant loss in sensitivity. By contrast, in specifically ^13^C-labeled proteins, the one-bond ^13^C–^13^C couplings are eliminated and the evolution period can be kept quite short due to the narrow frequency range of the aromatic region of the ^13^C spectrum. We tested both TROSY and PEP-HSQC approaches with either constant-time or non-constant time evolution periods on Gal3C at different global correlation times (*τ*_c_) by varying the temperature (Supplementary Fig. S1). The non-constant time PEP-HSQC experiment is more sensitive than the TROSY up to a global correlation time of about 13 ns (corresponding to a molecular weight of approximately 16 kDa at 5 °C), but the TROSY version results in narrower linewidths, as expected. We note that the inherent reduction in sensitivity that results from 1- or 2-^13^C_1_-glucose labeling (50 % incorporation) is essentially compensated for by the increase in sensitivity that results from the shortened *t*_1_ evolution period of the non-constant time experiment. Thus, aromatic ^1^H–^13^C correlation spectra of medium-sized and ^13^C_1_-glucose-labeled proteins have comparable sensitivities to those obtained using constant-time TROSY spectroscopy on uniformly ^13^C-enriched samples (Pervushin et al. 1998), thereby making the former samples very well suited also for other purposes than relaxation studies.

### Longitudinal relaxation optimization

In L-optimized spectroscopy (Pervushin et al. 2002), ^1^H spins not used for polarization transfer and detection are maintained close to equilibrium (i.e. magnetization along +z) throughout the experiment, including the relaxation, *t*_1_ evolution, and acquisition periods. This unperturbed “thermal bath” leads to efficient relaxation of the detected protons, as is well illustrated by comparing the effective relaxation rates in selective versus non-selective inversion recovery experiments (Cavanagh et al. 2007). Thus, L-optimization enables the use of shorter recycle delays and hence improved sensitivity in terms of signal-to-noise (S/N) per unit time. L-optimization is achieved using shaped, frequency-selective pulses to control the aromatic ^1^H spins separately from the water and aliphatic ^1^H spins. As described in more detail below, this level of control is critical in any type of quantitative experiment that starts from a non-equilibrium state and involves variable relaxation delays.

**Fig. 2:**
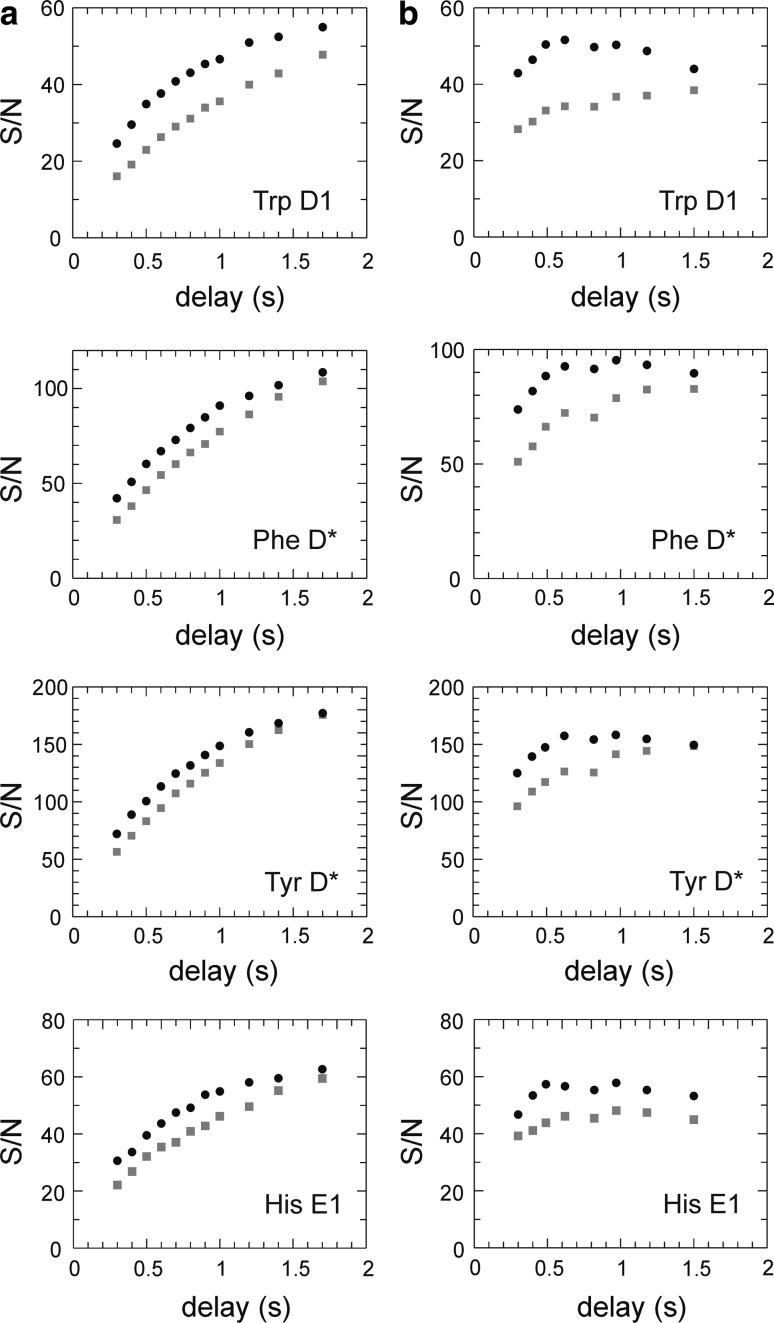
Sensitivity (signal-to-noise ratio, S/N) versus length of the recovery delay for L-optimized (*black circles*) and non-L-optimized (*grey squares*) versions of the *R*_1_ pulse sequence. Representative data are shown for different aromatic ^13^C sites (Trp181 Cδ1, Phe163 Cδ*, Tyr247 Cδ*, and His208 Cε1). **a** data acquired with a constant number of transients. **b** data acquired with a constant total experiment time. An optimal recovery time was estimated to 0.6 s for L-optimized and 1.5 s for non-optimized versions (*right-hand column*). The average gain in S/N for L-optimization is 35 % (Trp Cδ1), 10 % (Phe Cδ*), 10 % (Tyr Cδ*), and 25 % (His Cε1). Similar results are obtained for the *R*_2_ experiment

We investigated the benefits of L-optimization by comparing with non-L-optimized pulse sequences. Figure 2a shows the signal-to-noise ratio (S/N) as a function of recovery delay in the *R*_1_ experiment. To find the optimal recovery delay with respect to S/N, we repeated both the L-optimized and non-L-optimized experiments using the same total experimental time for each measured point (Fig. 2b). The optimal recovery time for L-optimized experiments of aromatics was determined to be 0.6 s for Gal3C. The average gain in S/N achieved with L-optimization was 10 % for Phe and Tyr Cδ*, 25 % for His Cδ2 and Cε1, and 35 % for Trp Cδ1. The additional increase in S/N observed for Trp and His is most probably caused by the presence of exchangeable protons vicinal to the ^13^C sites in question; exchange of these protons for water protons with near-equilibrium magnetization provides an efficient relaxation mechanism for the protons attached to the labeled carbons. The increased S/N is particularly useful in the context of 1-^13^C_1_- or 2-^13^C_1_-glucose labeling, which restricts the level of ^13^C incorporation to 50 %, and consequently limits the overall sensitivity of these samples. Phe and Tyr usually have inherently higher sensitivity due to the presence of two chemically identical carbons that contribute to the same peak intensity whenever the two resonances are averaged by rapid ring flips. Thus, L-optimization preferentially augments those side chains (Trp and His) that have the lowest inherent sensitivity, thereby making the signal intensities from the different aromatic sites more uniform on average. This result contrasts with what is observed for ^1^H–^15^N correlated experiments, where L-optimization typically leads to a less uniform distribution of signal intensities (Pervushin et al. 2002).

**Fig. 3:**
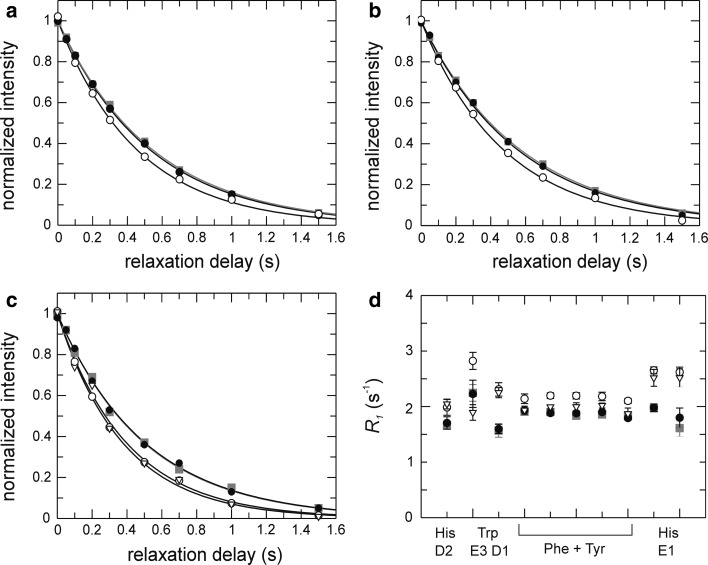
Dependence of *R*_1_ on the treatment of the ^1^H magnetization during the variable relaxation delay. **a**–**c** Representative relaxation decays (**a** Phe163 Cδ*; **b** Phe190 Cδ*; **c** His208 Cε1) obtained using pulse sequences without (*grey squares*) or with L-optimization using selective i-SNOB-5 (*black circles*) or non-selective (*open circles*) ^1^H inversion pulses during the relaxation delay. Panel **c** includes data obtained using rectangular ^1^H inversion pulses in combination with water flip-back (*open triangles*). **d** *R*_1_ rate constants obtained with the different implementations for different types of aromatic side chains, from *left* to *right*: His222 Cδ2, Trp181 Cε3, Trp181 Cδ1, Phe198 Cδ*, Phe149 Cδ* + Phe159 Cδ* + Tyr118 Cδ* + Tyr221 Cδ*, Phe163 Cδ*, Phe192 Cδ*, Phe209 Cδ*, His158 Cε1, and His217 Cε1. The non-L-optimized experiment utilized a recycle delay of 3.8 s

We next verified that the L-optimized pulse sequences yield accurate relaxation rates. Figure 3a–c show a comparison of *R*_1_ decay curves acquired using L-optimized and standard (i.e. non-L-optimized) experiments acquired with a 3.8 s recovery delay to ensure that the aromatic ^1^H magnetization has relaxed back to equilibrium. The relaxation decays and fitted relaxation rate constants (Fig. 3d) obtained using the L-optimized experiment are identical to those from the standard experiment, provided that ^1^H decoupling during the relaxation delay is applied as selective pulses on the aromatics. Non-selective ^1^H 180° pulses (in the context of L-optimization) lead to faster decays and higher apparent ^13^C *R*_1_ values. This result is a consequence of the intermittent inversion of water and aliphatic ^1^H magnetization between +z and −z during the relaxation period, which makes the state of the water/aliphatic magnetization at the end of each transient dependent on the length of the relaxation period, which in turn influences the relaxation of aromatic ^1^H spins, and hence the magnetization available for polarization transfer at the beginning of each ‘scan’. The outcome is that the intensity of the monitored ^13^C resonances will depend on the length of the relaxation period, thereby affecting the ^13^C intensity decay curves that define the measured (apparent) relaxation rate constants; similar observations have been reported recently for ^15^N relaxation experiments (Chen and Tjandra 2011). Therefore, it is critical in L-optimized experiments to maintain the ^1^H magnetization of water and aliphatics along +z throughout the relaxation delay in order to ensure accurate relaxation measurements.

The results shown in Fig. 3c–d further reveal the extent to which the water and aliphatic magnetizations contribute to the increased relaxation rate of the different aromatic ^1^H spins achieved with L-optimization. We carried out *R*_1_ experiments in which selective ^1^H pulses were applied to the water resonance only, thereby restricting the origin of the L-optimization effect to water spin pool. By applying non-selective ^1^H 180° pulses during the relaxation delay we observe the type of artefacts described in the section above, but only to the extent that the relaxation of the aromatic protons is influenced by the state of the water magnetization. In this experiment, we observe artificially increased *R*_1_ rates for atoms vicinal to sites with exchangeable protons, like Cδ2 and Cε1 in His and Cδ1 in Trp (Fig. 3c–d). By contrast, rates comparable to or slightly higher than those obtained from the standard or L-optimized experiments were observed for other sites, reflecting the signficantly reduced contribution from water in driving the relaxation of these spins. Thus, the water magnetization contributes to L-optimization mainly for ^1^H spins situated nearby protons that exchange with water, in agreement with the higher intensity gain observed for histidines and tryptophanes (cf. Fig. 2).

### L-optimized R_2_ experiment

**Fig. 4:**
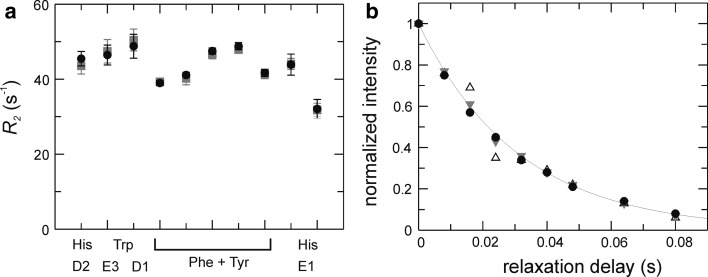
*R*_2_ CPMG in-phase relaxation experiment. **a** *R*_2_ rate constants for different types of aromatic ^13^C sites determined using L-optimized (*black circles*) and non-L-optimized (*grey squares*) experiments. **b** Relaxation decays for His ^13^Cε1 obtained with τ_b_ = 1.623 ms (tuned to ^1^ *J*_HC_ = 154 Hz) and the purge gradient G4 included (*filled circles*), or without gradient G4 and τ_b_ = 1.623 ms (*open triangles*) or τ_b_ = 1.2 ms (tuned to ^1^ *J*_HC_ = 208 Hz, *grey triangles*)

The *R*_2_ relaxation experiment can be implemented in a straightforward fashion within the general framework presented above for the *R*_1_ experiment (Fig. 1b). Again, it is advisable that the ^1^H 180° pulses during the relaxation delay are selective for the aromatics in order to achieve the full benefits of L-optimization. However, the generally shorter relaxation periods needed to sample transverse relaxation decays serve to limit the adverse effects on the ^13^C relaxation measurements that might arise due to differential recovery of water/aliphatic magnetization as a function of the length of the relaxation period. Indeed, in the present case, L-optimized and non-L-optimized experiments yield identical *R*_2_ rates, within errors (Fig. 4a).

The one-bond ^1^H–^13^C coupling constants in aromatic side chains depend on the chemical structure as follows: ^1^ *J*_HC_ = 155 Hz for sites in 6-membered rings; ^1^ *J*_HC_ = 185 Hz for sites in 5-membered rings with a single directly attached nitrogen, i.e. Cδ2 in His and Cδ1 in Trp; ^1^ *J*_HC_ = 205 Hz for sites in 5-membered rings with two directly attached nitrogens, i.e. Cε1 in His. The non-uniform one-bond ^1^H–^13^C coupling constants require that non-refocused anti-phase magnetization be purged using a strong gradient pulse (G4 in Fig. 1b). In the absence of the purging gradient and with the refocusing delay optimized for smaller coupling constants (155–185 Hz), the residual anti-phase term gives rise to oscillations in the relaxation decay, as demonstrated for Cε1 in His (Fig 4b). Of course, *R*_2_ rates can alternatively be measured in separate experiments that employ refocusing delays matching the different values of ^1^ *J*_HC_; this approach also serves the purpose to optimize the sensitivity for each class of sites.

**Fig. 5:**
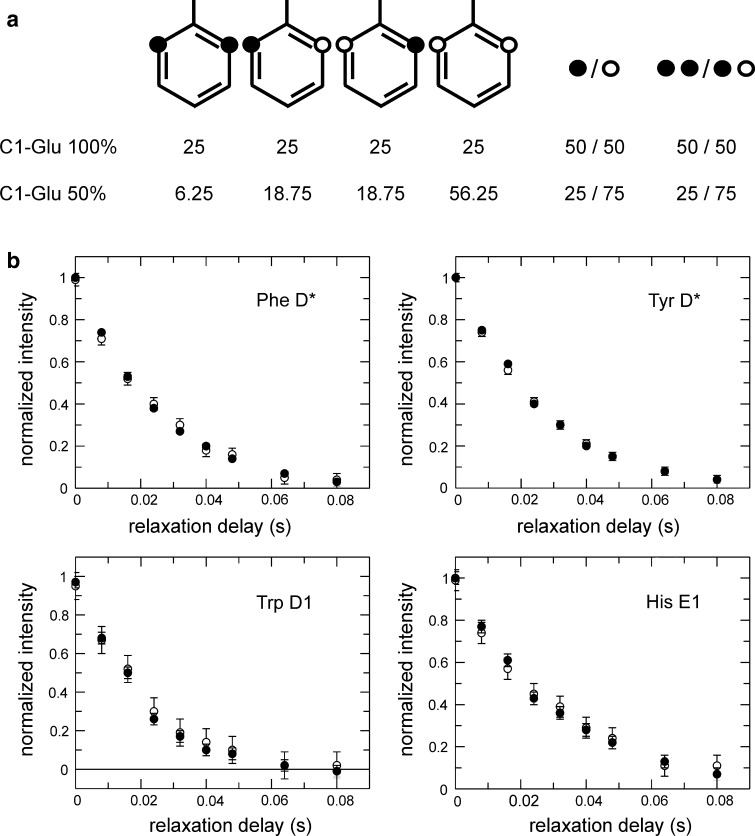
Influence of two-bond ^13^C–^13^C *J* couplings on measured *R*_2_ relaxation decays. **a** ^13^C incorporation pattern in a Phe side chain resulting from labeling with 1-^13^C_1_-glucose. *Black circles* represent ^13^C-labeled positions while *open circles* represent ^12^C. The *symbols* to the *right* of the aromatic rings show the net percentages of labeled or unlabeled sites (labeled/unlabeled), and the percentages of ^13^C sites with or without a two-bond neighbor (labeled–labeled/labeled–unlabeled). Labeling using 100 % 1-^13^C_1_-glucose yields 50 % ^13^C incorporation in the Cδ positions, and 50 % of these ^13^Cδ have a ^13^C-labeled two-bond neighbour. Labeling using 50 % 1-^13^C_1_-glucose + 50 % ^12^C_6_-glucose reduces the relative number of labeled carbons, as well as that of labeled two-bond neighbours, to 25 %. **b** Representative *R*_2_ relaxation decays for different aromatic side chains in samples labeled using 100 % 1-^13^C_1_-glucose (*filled circles*) or 50 % 1-^13^C_1_-glucose + 50 % ^12^C_6_-glucose (*open circles*)

While 1-^13^C_1_- and 2-^13^C_1_-glucose labeling effectively eradicates directly neighboring ^13^C sites in the aromatic rings, 50 % of the labeled carbons still have another ^13^C nucleus two bonds away. The two-bond scalar coupling is expected to be small, ^2^ *J*_CC_ ≈ 2–7 Hz (Kaski et al. 1996; Witanowski et al. 2007), but is practically impossible to refocus in most cases due to the narrow spectral range covered by the two resonances. We investigated the influence of the ^2^ *J*_CC_ coupling on the measured *R*_2_ rates by comparing the results obtained from two different samples labeled using either 100 % 1-^13^C_1_-glucose or 50 % 1-^13^C_1_-glucose + 50 % ^12^C_6_-glucose, resulting in 50 % or 25 %, respectively, of the observed carbons having a two-bond coupling partner (Fig. 5a). The resulting *R*_2_ decays are identical within errors (Fig. 5b), thus verifying that two-bond ^13^C-^13^C couplings do not affect ^13^C *R*_2_ measurements to any appreciable extent.

### {^1^H}–^13^C *NOE* experiment

**Fig. 6:**
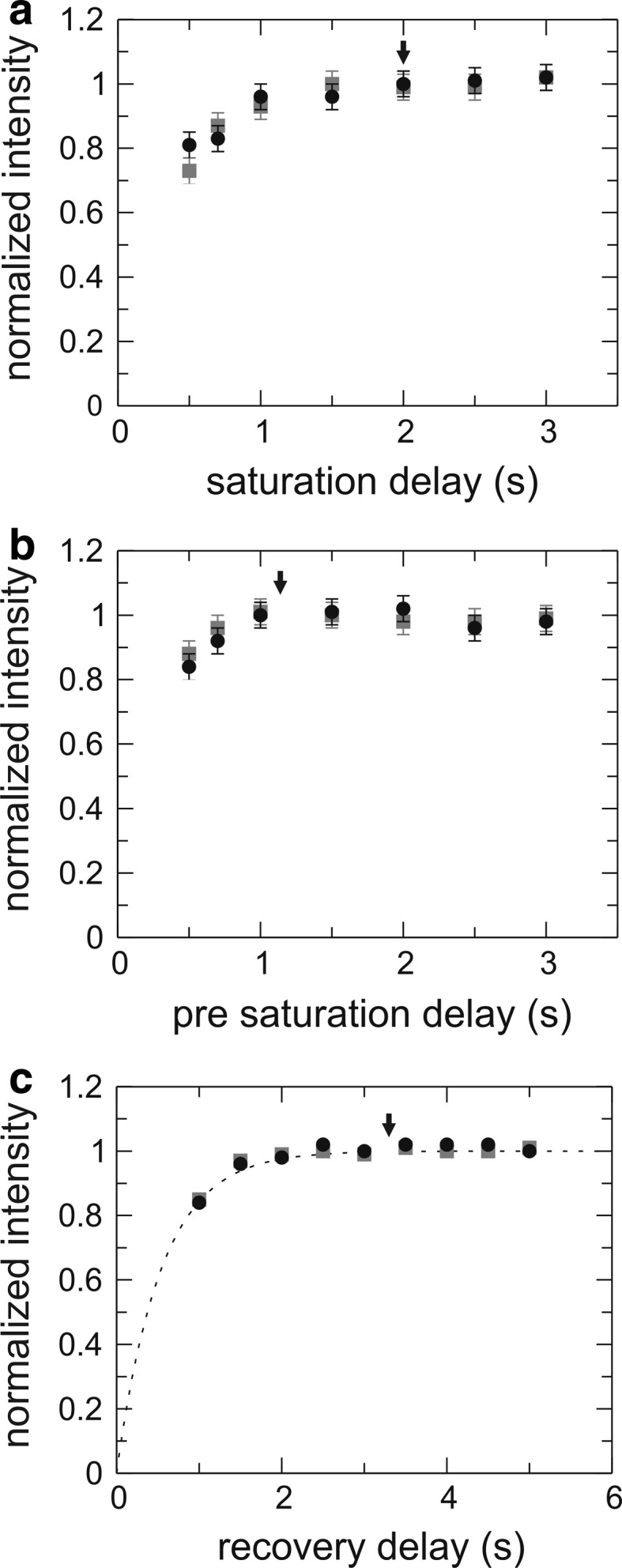
Intensity build-up in the aromatic {^1^H}–^13^C *NOE* experiment. **a** Difference intensity (saturation experiment − reference experiment) of the {^1^H}–^13^C *NOE* experiment shown as a function of ^1^H saturation time for the pulse sequences that correspond to the L-optimized (*black circles*) and non-L-optimized (*grey squares*) versions, using a constant pre-saturation delay of 4 s. **b** Difference intensity as a function of pre-saturation delays using a ^1^H saturation time of 2 s. **c** Intensity originating from ^13^C excitation (reference experiment) as a function of the recovery delay. Optimal delays are indicated with *black arrows*: the ^1^H saturation period is 2 s, and the pre-saturation delay is 1.3 s. The resulting recovery delay of 3.3 s equals approximately 6/*R*_1_, ensuring that the ^13^C magnetization has completely returned to equilibrium. The data represent the summed intensity of four residues: Phe149 Cδ* + Phe159 Cδ* + Tyr118 Cδ* + Tyr221 Cδ*

The {^1^H}–^13^C *NOE* saturation and reference experiments were optimized in a straightforward fashion by finding the shortest possible saturation and recycle periods that still provide full magnetization transfer and recovery. Since the length of the *NOE* reference experiment is governed by the recovery of the heteronucleus (^13^C in the present case), L-optimization offers little or no advantage for a medium-sized protein like Gal3C, as shown in Fig. 6. Still, selective ^1^H pulses for proton saturation are beneficial in order to achieve equal water suppression in both the saturation and reference experiments. For Gal3C, a ^1^H saturation period of 2 s is sufficient to achieve complete *NOE* transfer (Fig. 6a). This value is in agreement with the data shown in Fig. 2a, demonstrating that the chosen length of the saturation period is close to 6·*T*_1_ for the aromatic protons. As shown in Fig. 6b, a pre-saturation delay of 1.3 s guarantees full recovery of the ^1^H magnetization. The recycle delay of the reference experiment is set to 3.3 s (approximately 6·*T*_1_ for aromatic ^13^C spins), which ensures that the ^13^C magnetization has relaxed back to equilibrium (Fig. 6c).

### Vicinal and remote protons do not affect the relaxation of aromatic ^13^C spins

**Fig. 7:**
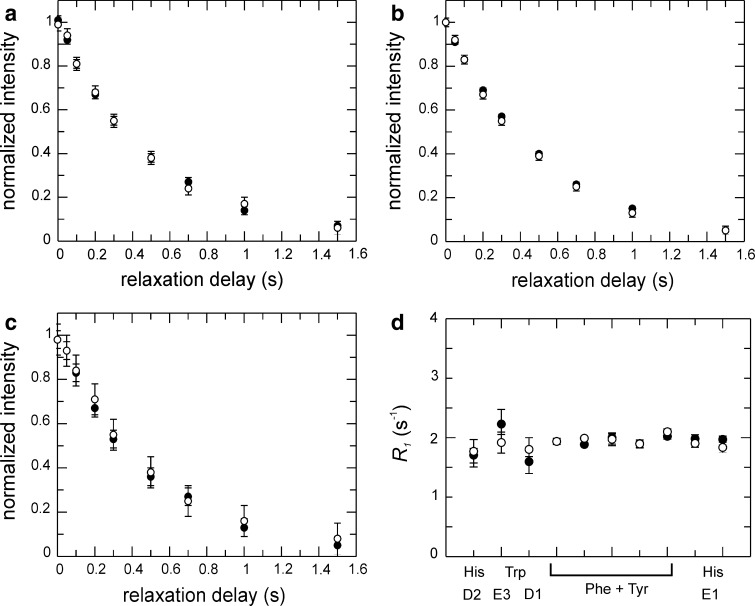
Comparison of ^13^C *R*_1_ relaxation in partially deuterated and non-deuterated samples. **a**–**c** Representative *R*_1_ relaxation decays measured for two Phe residues (**a**–**b**) and one His (**c**) on a partially deuterated sample (*open circles*) and non-deuterated sample (*black circles*). **d** Fitted *R*_1_ relaxation rate constants for all ^13^C sites characterized in Gal3C. The deuteration levels at the vicinal sites are estimated to 50–55 % for Phe and Tyr ^1^Hε, and 60 % for Trp ^1^Hζ3 (see Table S1)

The interpretation of the ^13^C relaxation rates in terms of models describing the motion of the aromatic rings is greatly simplified if the relaxation is dominated by the CSA and dipolar interaction with the covalently attached proton (bond length 1.08 Å). We established that there is no detectable influence from ^1^H spins located near the aromatic ^1^H–^13^C moieties of interest. The vicinal protons are expected to be the closest ones, at a distance of 2.14 Å in the case of Phe and Tyr. The dipolar interaction with the vicinal protons is reduced by a factor of approximately (1.08/2.14)^6^ = 1.7 %, compared to that of the covalently attached proton; consequently, relaxation effects due to vicinal or other neighboring protons are expected to be small. We verified the theroretical prediction by comparing ^13^C relaxation rates measured on samples that were either fully protonated or partially deuterated to a level of 50–60 % at the vicinal sites (Table S1) and approximately 50 % on average. As shown in Fig. 7, there are no discernable differences in ^13^C relaxation between these two samples. Thus, we conclude that relaxation measurements on specifically ^13^C labeled aromatic side chains are unaffected by remote protons.

## Conclusions

We have shown that L-optimization increases the sensitivity (defined in terms of signal-to-noise per unit time) of ^13^C relaxation experiments for aromatic side chains by at least 10–35 %. This level of S/N enhancement was attained for a relatively small protein of 16 kDa at a static magnetic field strength of 11.7 T. Significantly higher S/N enhancement are expected for bigger proteins and higher static magnetic field strengths, which both result in slower ^1^H *R*_1_ relaxation rates and therefore makes L-optimization increasingly advantageous. Proteins larger in size than approximately 25–30 kDa (at 25 °C) benefit further from TROSY-based ^13^C chemical shift evolution. Furthermore, we demonstrate that the relaxation measurements are not significantly affected by potential complications due to residual two-bond ^13^C–^13^C scalar couplings or dipolar interactions with neighboring ^1^H spins. Precise control of the water and aliphatic ^1^H magnetizations enables accurate measurements of ^13^C relaxation rate constants using L-optimized pulse sequences.

## Electronic supplementary material

Below is the link to the electronic supplementary material.

Supplementary material 1 (PDF 770 kb)
