# Supplementary material for: 13C relaxation experiments for aromatic side chains employing longitudinal- and transverse-relaxation optimized NMR spectroscopy
Source: J Biomol NMR. 2012 Jul 3;53(3):181–90. doi: 10.1007/s10858-012-9650-5 (PMC3405241; doi:10.1007/s10858-012-9650-5)
Supplement: Supplementary file 1 — Supplementary material 1 (PDF 770 kb) [file 10858_2012_9650_MOESM1_ESM.pdf]

Supplementary Information

^{13}C relaxation experiments for aromatic side chains employing longitudinal- and transverse-relaxation optimized NMR spectroscopy

Ulrich Weininger, Carl Diehl, Mikael Akke*

Department of Biophysical Chemistry, Center for Molecular Protein Science, Lund University, P.O. Box 124, SE-22100 Lund, Sweden

*Correspondence should be addressed to
Mikael Akke
phone: +46-46-2228247
mobile: +46-76-8585708
e-mail: mikael.akke@bpc.lu.se

Fig. S1

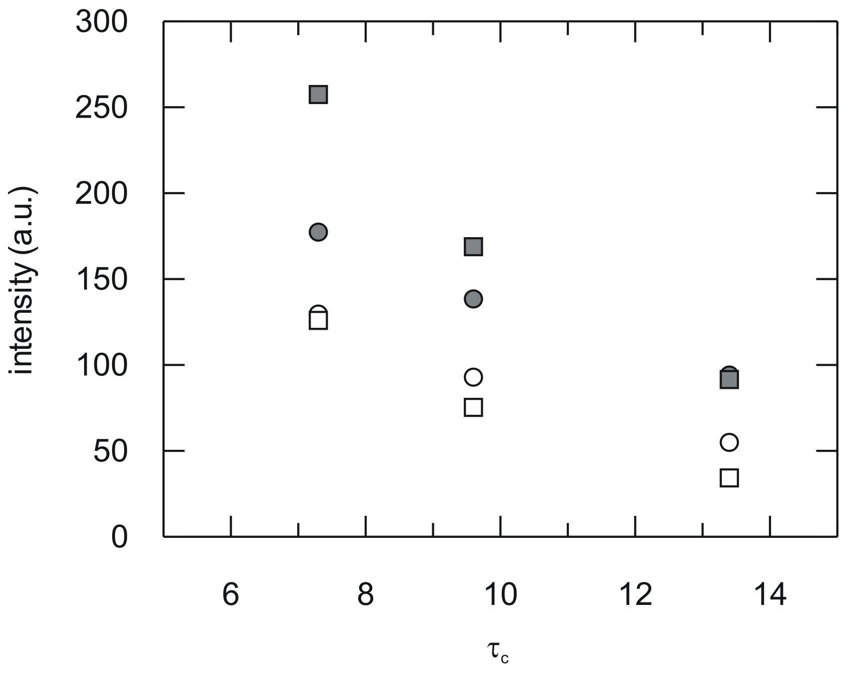

Figure S1: Representative example of cross-peak intensity in ^{1}H-^{13}C PEP-HSQC (squares) or ^{1}H-^{13}C TROSY (circles) experiments acquired with different rotational correlation times (τ_C) by varying the temperature between 5–28 °C. Open symbols represent experiments employing constant-time (17.6 ms) t_1 evolution, while filled symbols represent non-constant time experiments. The rotational correlation time was determined at 28 °C based on ^{15}N relaxation experiments [Diehl et al., (2010) *J. Am. Chem. Soc.* 132, 14577–14589], and extrapolated to the lower temperatures using the Stokes-Einstein relationship.

Table S1

Protonation levels for different positions in aromatic side chains in Gal3C expressed in 60% D_2O + 40% H_2O.

Amino acid	position	I_D/I_H (%)	$p_{solvent}$ (%)
Phe	D*	80	33
	E*	55	75
Tyr	D*	70	50
	E*	50	83
His	D2	55	75
	E1	80	33
Trp	D1	80	33
	E3	100	0
	Z3	60	66
	Z2	70	50

Intensities where compared in ^1H–^{13}C HSQC spectra recorded on Gal3C expressed in ^{15}N NH_4Cl and 1-$^{13}C_1$-glucose or 2-$^{13}C_1$-glucose dissolved in either 100% H_2O or 60% D_2O + 40% H_2O. The relative intensities of the two samples were calibrated based on the intensities in the corresponding ^1H–^{15}N HSQC spectra. The percentage of aromatic hydrogens exchanged for solvent hydrogens was calculated as

$$p_{solvent} = \frac{I_H - I_D}{0.6 I_H},$$

where I_H and I_D are the intensities in the samples expressed in 100% H_2O and 60% D_2O + 40% H_2O, respectively.
